# Automatic segmentation of male pelvic floor soft tissue structures for anatomical simulation and morphological assessment in lower rectal cancer surgery

**DOI:** 10.1007/s10151-025-03218-z

**Published:** 2025-10-08

**Authors:** Y. Aisu, T. Okada, Y. Itatani, A. Masuo, R. Tani, K. Fujimoto, A. Kido, A. Sawada, Y. Sakai, K. Obama

**Affiliations:** 1 Department of Surgery, Kyoto University Graduate School of Medicine, 54 Shogoin Kawaharacho, Sakyo-ku, Kyoto, Kyoto 606-8507 Japan; 2 Department of Surgery, Japanese Osaka Red Cross Hospital, Osaka, Japan; 3 Department of Diagnostic Radiology and Nuclear Medicine, Kyoto University Graduate School of Medicine, Kyoto, Japan; 4 Department of Radiology, University of Toyama, Toyama, Japan; 5 Department of Urology, Faculty of Medicine, University of Miyazaki, Miyazaki, Japan

**Keywords:** Pelvic floor, Anatomy, Image segmentation, Artificial intelligence

## Abstract

**Background:**

Pelvic anatomy is a complex network of organs that varies between individuals. Understanding the anatomy of individual patients is crucial for precise rectal cancer surgeries. Therefore, developing technology that can allow visualization of anatomy before surgery is necessary. This study aims to develop an auto-segmentation model of pelvic structures using AI technology and to evaluate the accuracy of the model toward preoperative anatomical understanding.

**Methods:**

Data were collected from 63 male patients who underwent 3D MRI during a preoperative examination for colorectal and urogenital diseases between November 2015 and July 2019 and from 11 healthy male volunteers. Eleven organs and tissues were segmented. The model was developed using a threefold cross-validation process with a total of 59 cases as development data. The accuracy was evaluated with the separately prepared test data using dice similarity coefficient (DSC), true positive rate (TPR), and positive predictive value (PPV) by comparing AI-segmented data with manual-segmented data.

**Results:**

The highest value of DSC, TPR, and PPV were 0.927, 0.909, and 0.948 for the internal anal sphincter (including the rectum), respectively. On the other hand, the lowest values were 0.384, 0.772, and 0.263 for the superficial transverse perineal muscle, respectively. While there were differences among organs, the overall quality of automatic segmentation was maintained in our model, suggesting that the morphological characteristics of the organs may influence the accuracy.

**Conclusions:**

We developed an auto-segmentation model that can independently delineate soft-tissue structures in the male pelvis using 3D T2-weighted MRIs, providing valuable assistance to doctors in understanding pelvic anatomy.

**Supplementary Information:**

The online version contains supplementary material available at 10.1007/s10151-025-03218-z.

## Introduction

In recent years, novel approaches and devices such as transanal total mesorectal excision (TaTME) and surgical robots have been actively pursued for rectal cancer surgery [1–4]. Magnified and high-resolution views have led to increased demand by colorectal surgeons for a more detailed understanding of the structures of the deep pelvis. The anatomy of the pelvic cavity is complex and consists of various organs such as muscles, bones, rectum, urogenital organs, nerves, and blood vessels. Each structure varies from person to person, and identifying the pelvic floor muscles before surgery is a challenging task even for experienced surgeons. Urethral injury, the most concerning complication encountered during TaTME, is attributed to insufficient comprehension of the anatomy and recognition of key landmarks [5–7]. Spatial perception of pelvic anatomy using preoperative images requires extensive knowledge of the anatomy and significant effort. Therefore, a three-dimensional (3D) pelvic floor diagram will contribute to a preoperative understanding of the pelvic anatomy and improve landmark recognition during surgery. It also facilitates preoperative simulation and intraoperative navigation [8].

Considering postoperative anorectal function in patients with lower rectal cancer is important, as patients who undergo low anterior resection (LAR) often develop LAR syndrome (LARS), an anorectal dysfunction after LAR. In previous reports, the assessment of anorectal function mainly included morphological information on the pelvic floor muscles, particularly the puborectalis muscle [9–12]. The volume of these muscles has been suggested to be related to LARS [12]. However, evaluating this manually from preoperative images is time-consuming, and developing an automatic evaluation technique with high accuracy is demanding.

In this study, we developed a high-speed artificial intelligence (AI) model that can automatically generate precise 3D anatomical diagrams of the pelvic organs from 3D T2-weighted magnetic resonance imaging (MRI) of the pelvis.

## Methods

### Collecting MRI data

**Fig. 1:**
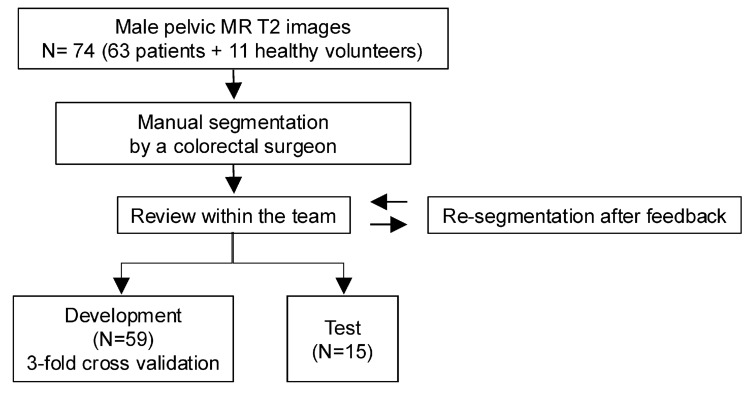
Overview of the training method

A total of 74 male cases were enrolled in this study (Fig. 1). Among these cases, 63 consecutive patients underwent 3D MRI at Kyoto University Hospital as a preoperative examination for colorectal and urogenital diseases between November 2015 and July 2019, and 11 were healthy volunteers. These data did not include cases with strong artifacts due to artificial joints, significant organ displacement due to large tumors, organ loss after surgery, imaging ranges not including the anal margin, and low-quality images due to excessive body motion. The Ethical Review Board of Kyoto University Hospital approved this study (R2119, R2095).

### 3D T2-weighted MRI protocol

All MR scans were performed using 3.0 T MR scanners (MAGNETOM Skyra or MAGNETOM Prisma, Siemens Healthcare, Erlangen, Germany) with an 18-channel phase-array body coil. The MRI protocol included sagittal T2-weighted turbo spin echo (TSE), oblique axial thin-section T2-weighted TSE, and oblique axial multi-b single-shot echo-planar-imaging DWI sequence. The parameters of the 3D T2-weighted TSE sequence used in this study were as follows: TR = 1800 ms; TE = 260 ms; parallel imaging factor = 2; voxel size = 1.0 × 1.0 × 1.0 mm; field of view = 260 mm; scan matrix = 256 × 240; slice thickness = 1.0 mm; phase over-sampling = 10%; number of sections = 128–144; bandwidth (Hz/pixel) = 814. No bowel preparation or air insufflation was performed, although intramuscular antispasmodic agents (scopolamine butyl bromide, 20 mg IV) were routinely administered.

### Image annotation

Eleven male pelvic anatomy structures obtained from MRI were manually delineated by an experienced colorectal surgeon using Amira (Thermo Fisher Scientific, Waltham, MA, USA); the 11 tissues were (1) external anal sphincter (subcutaneous part), (2) external anal sphincter (superficial and deep part), (3) internal anal sphincter (including rectal wall and lumen), (4) puborectalis muscle, (5) coccygeal muscle, (6) pubococcygeal and iliococcygeal muscle, (7) bulbospongiosus muscle, (8) urethral sphincter, (9) superficial transverse perineal muscle, (10) urethra, and (11) prostate. In this study, we confirmed the anatomical structures in advance on the basis of our previous cadaveric study and began creating training data after sharing a common understanding among the team members [13]. These structures are delineated from the superior margin of the prostate to the anal verge. 3D T2-weighted MRI allowed structures to be delineated from multiple angles. The internal sphincter muscle and rectal lumen were included in the same delineation area because the rectal lumen was almost completely crushed inside the anal canal. The urethra and bulbospongiosus muscles were delineated in the area affected by surgery. After delineation, two surgeons and two radiologists reviewed the segmented labels and provided feedback about corrections. The data agreed upon by all the members were used as development and test data (Fig. 1). In this study, we employed a U-Net-based deep learning model with a depth of seven, which corresponds to the number of downsampling and upsampling steps, in cooperation with Ziosoft Inc. (Tokyo, Japan) [14]. Input images were resized to 512 × 512 pixels and normalized to a signal range of [0,1]. The loss function was multi-label cross-entropy loss, and the Adam optimizer was used with a learning rate of 0.001. To mitigate overfitting, we applied data augmentation techniques. Specifically, an operation that erased 5% of the image area in a rectangular shape at a random location with a probability of 30% was employed. The model was developed using a threefold cross-validation process with a total of 59 cases as development data. The final model was an ensemble of the best-performing epoch (in terms of validation performance) from each of the three folds.

### Performance evaluation

The accuracy of the developed model was evaluated using the dice similarity coefficient (DSC), true positive rate (TPR), and positive predictive value (PPV) by comparing AI-segmented data to manually segmented data (test data) in 15 cases that were separately prepared from the development dataset. Furthermore, the usability of the algorithm was assessed by computing the time required for automatic segmentation.

## Results

### Patient characteristics

Patient backgrounds are presented in Table 1. To enhance the generalizability of the model, we collected MRI data from patients from various backgrounds, including those from colorectal and urological departments and healthy volunteers. Two patients with bladder cancer were included in the test group. Because we did not delineate the bladder in this study, this should not affect the outcomes. Other patient background factors did not differ between the two groups.

**Table 1:**
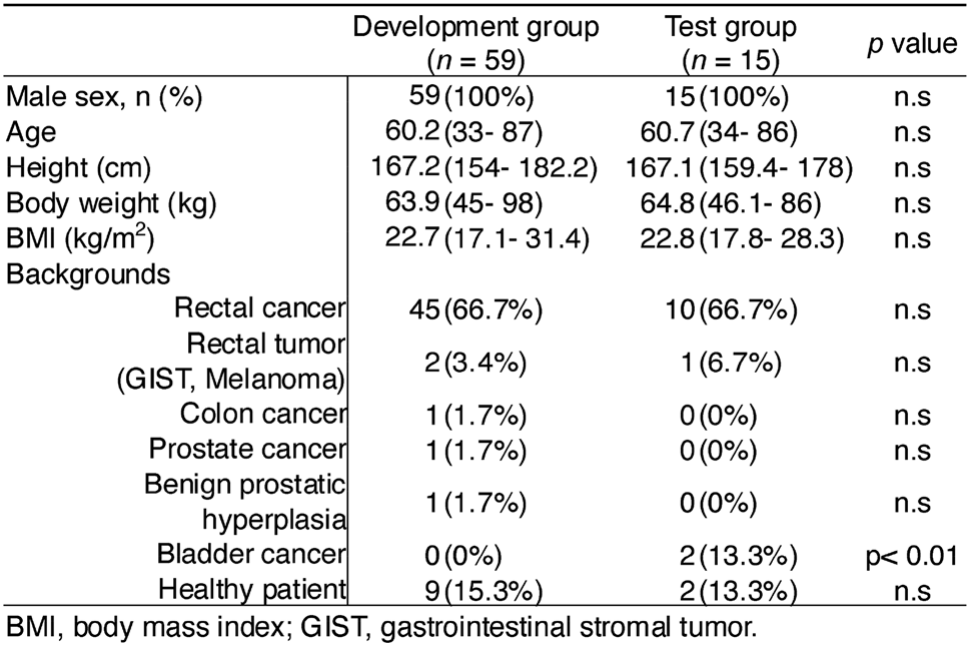
Patient characteristics

### Automatic segmentation model for pelvic soft tissue anatomy

**Fig. 2:**
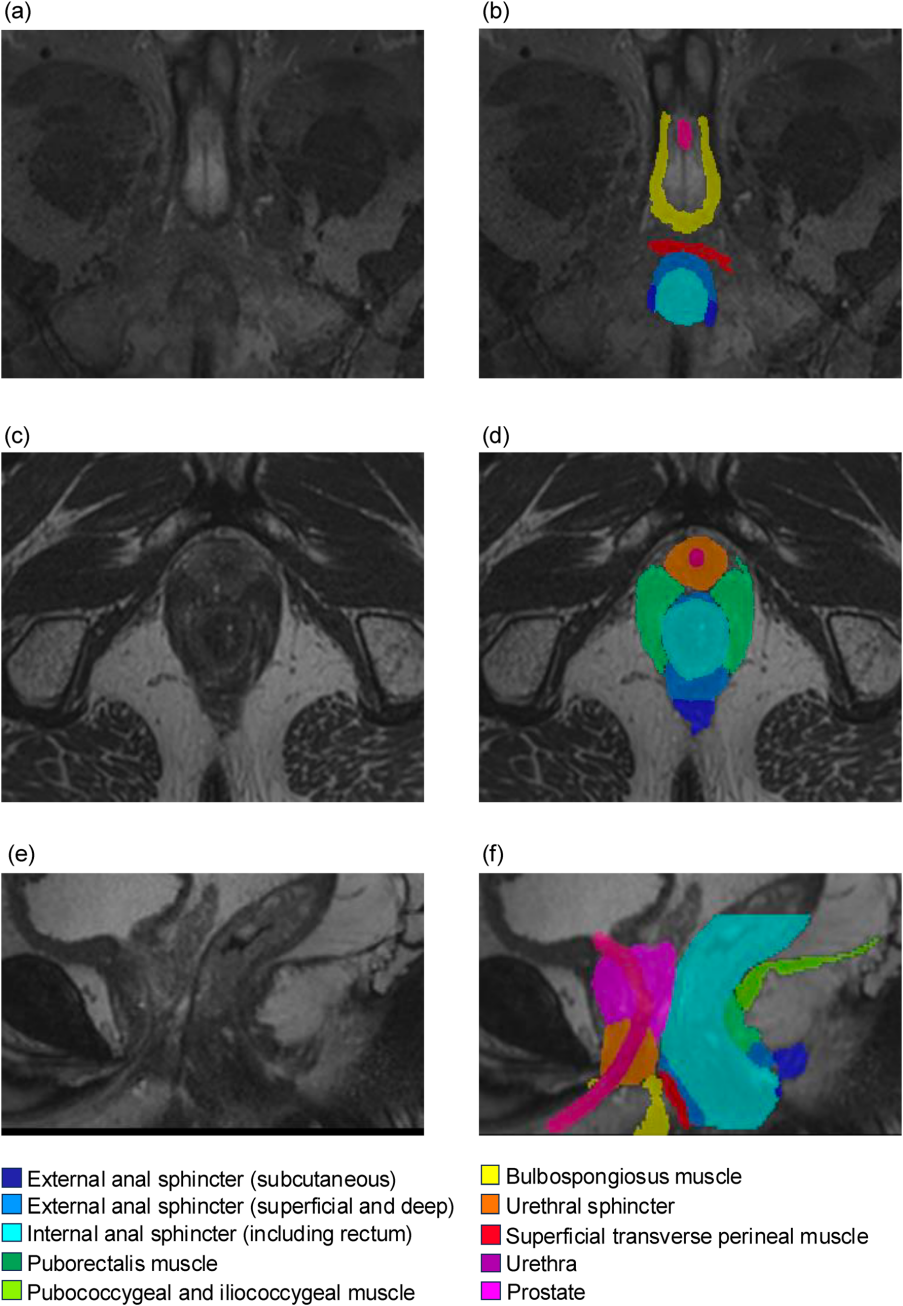
Segmentation label created by the artificial intelligence-based algorithm. **a** Axial T2 MRI image at the level of anal canal. **b** AI segmentation labels overlaid on **a**. **c** Axial T2 MRI image at the level of puborectalis muscle. **d** AI segmentation labels overlaid on **c**. **e** Midsagittal T2 MRI image. **f** AI segmentation labels overlaid on **e**

**Fig. 3:**
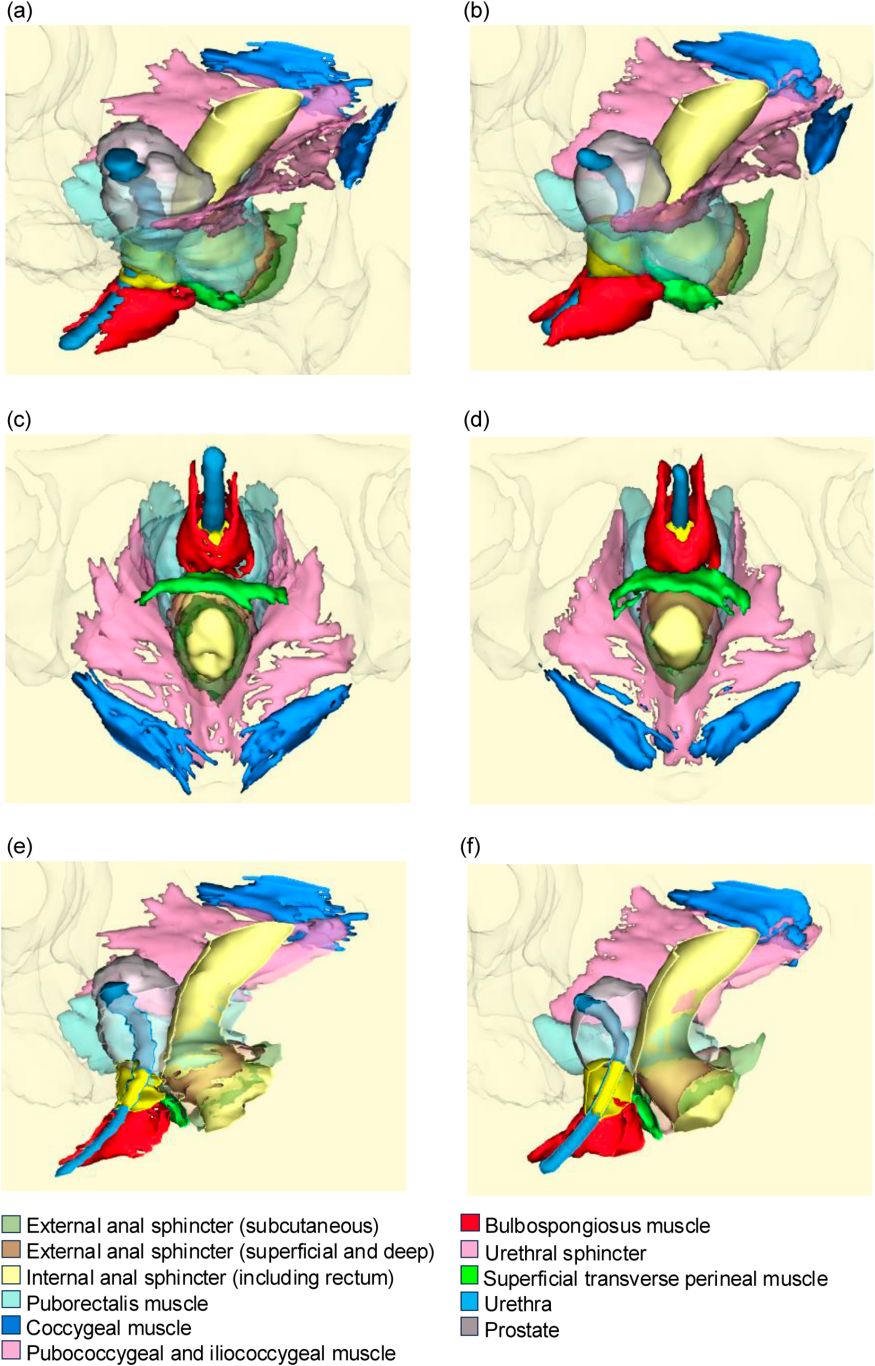
Three-dimensional pelvic anatomical diagram generated by ground-truth segmentation label and artificial intelligence-based algorithm. **a** 3D diagram generated by ground-truth label. **b** 3D diagram generated by artificial intelligence-based algorithm. **c** Caudal view of **a**. **d** Caudal view of **b**. **e** Midsagittal section of **a**. **f** Midsagittal section of **b**

We developed an automatic segmentation model that could extract 11 pelvic structures. The inference time, which depends on the computer specifications, was 689 ± 76 s (mean ± SD) for the 15 examples in the test group. The technical specifications of the computer were as follows: CPU, Intel Xeon E5-2690 Dual; RAM, 48 GB; GPU, NVIDIA TITAN RTX; and VRAM, 24 GB. The output of the process was a 3D segmentation mask image that automatically produces a 3D image of the entire pelvis. Figure 2 shows an example of the output created by the model. The 3D image obtained using this software could be sectioned at any arbitrary point and rotated freely, aiding the comprehension of the anatomical layered structure (Fig. 3). This allows easy simulation of anatomical structures, even when approaching from the anal side, such as with TaTME.

### Accuracy of the segmentation model

The accuracy of automatic segmentation of each part of the pelvic anatomy is presented in Table 2. The DSC for the internal anal sphincter (including the rectum) and the prostate was 0.927 and 0.865, respectively. The TPR for these structures was 0.909 and 0.829, with a PPV of 0.948 and 0.912, respectively. These results indicate that the automatic segmentation model developed in this study showed sufficient accuracy for massive spherical structures. In contrast, for other structures, the DSC ranged from 0.384 to 0.757, the TPR ranged from 0.514 to 0.923, and the PPV ranged from 0.263 to 0.783, which were less sufficient but still adequate compared to those of the two abovementioned structures. For a more detailed evaluation of segmentation performance, the confusion matrix is provided in Supplemental Table 1.

**Table 2:**
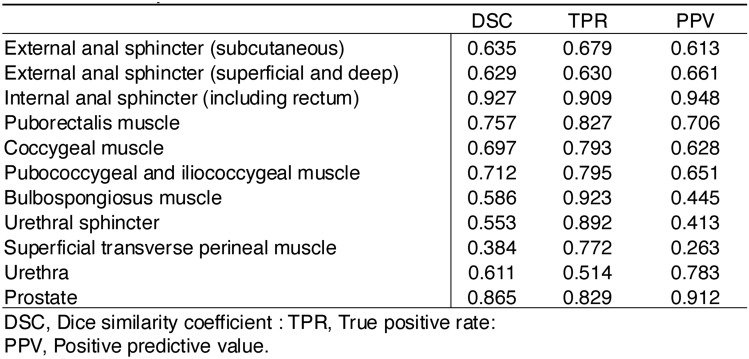
Performance of the automatic segmentation model

## Discussion

Using AI technology, we developed an automatic segmentation model that could generate a 3D diagram of the pelvic soft tissue anatomy from male pelvic 3D T2-weighted MRI. This model allows the creation of pelvic anatomy diagrams with high speed and sufficient precision. Although automatic detection of organs, blood vessels, nerves, bones, and tumors in the pelvic region has been reported previously [15–18], to the best of our knowledge, this is the first study to achieve automatic segmentation of the soft tissues of the male pelvic floor, including the individual muscles, rectum, and urogenital organs. We focused on male patients in this study because they have a narrow pelvis, a shorter distance between the rectum and genitourinary organs, and a higher risk of urethral injury during TaTME [5–7]. Our model provides valuable information regarding the pelvic anatomy of male patients, providing a valuable contribution to safe surgeries.

The boundaries between muscles are generally blurred even on 3D T2-weighted MRI, making it extremely difficult to distinguish each pelvic floor muscle. Therefore, the segmentation of individual pelvic floor muscles has not yet been attempted. For detailed and correct segmentation data, we comprehended the anatomical structure by performing a macro/microscopic cadaveric study of male pelvic anatomy [11], annotated the organs using 3D viewer software that allowed us to view images from multiple angles, reviewed the segmented labels, and provided feedback for corrections.

The influence of morphological variations on the precision of semantic segmentation is well known. In particular, objects that are spherical and large in volume are typically associated with higher accuracy, whereas objects that are flat and small in volume are often associated with lower accuracy [19]. A similar trend was observed in this study. The accuracy for massive spherical structures (such as the prostate and the internal anal sphincter, including the rectal lumen) was good and comparable with previous reports [20–22], whereas the accuracy for long planar structures (such as superficial transverse perineal muscle) did not yield favorable results compared to the massive spherical structures but still provided acceptable values. In long planar structures, minor deviations have a significant negative impact on accuracy [19]. The superficial transverse perineal muscle, with a low DSC of 0.384, is believed to be particularly affected by its thinness, making it barely observable in some cases, thereby amplifying the impact of any deviations. These results imply that this morphological diversity affected the accuracy of auto-segmentation. Because no prior reports exist about the automatic segmentation of pelvic floor soft tissues, a straightforward comparison of accuracy is not feasible. However, given the satisfactory accuracy levels achieved for massive spherical structures, it can be inferred that the overall quality of the automatic segmentation was preserved in our model. These findings suggest that the slightly lower accuracy observed for certain structures may be attributable to their morphological characteristics. Notably, there is still significant room for improvement in the segmentation accuracy of long planar structures.

The process of manually generating a 3D anatomical pelvic diagram from a 3D T2-weighted MRI is time-consuming and requires a detailed understanding of the pelvic anatomy. In this study, it took approximately 8 h per case to annotate 11 pelvic organs in the initial stages from approximately 150 slides in the axial plane; afterward, it was confirmed in the coronal and sagittal planes. This duration was reduced to approximately 3 h even at the plateau stage. In contrast, the developed model can generate precise anatomical pelvic diagrams in approximately 11 min, significantly alleviating the temporal and physical strain on the medical staff. In addition, with more high-performance computers, this duration can be further reduced. Furthermore, the volume of muscles involved in anal function can be easily assessed, and these data may be a new indicator for the evaluation of defecation function.

Advancements in autonomous robotic surgery (ARS) that integrate AI are gaining momentum globally [23]. Because autonomous vehicle navigation requires a base map, a 3D pelvic anatomical diagram specific to each patient would become an indispensable base map for ARS implementation. This study was the first attempt to create a 3D anatomical pelvic diagram, and the ability to swiftly acquire patient-specific diagrams could have a substantial impact on the ARS field in the future.

This study had several limitations. First, only male patients were included. Therefore, whether the application of our model to female patients will yield accurate results is uncertain. Additionally, the results cannot be guaranteed for cases with poor image quality owing to motion and artifacts, giant tumors, or organ defects, as these were not included in the training data. The model was trained using a dataset of MRI images from a single institution. This may limit the generalizability of the model to other settings. Further research is required to expand this adaptation and improve the model accuracy.

## Conclusion

We developed an AI-based algorithm to automatically segment male pelvic soft tissue structures from 3D T2-weighted MRI. Our model could segment the pelvic structures quickly and efficiently, assisting doctors in understanding pelvic anatomy.

## Supplementary Information

Below is the link to the electronic supplementary material.

Supplementary file1 (TIFF 946 KB)

## Data Availability

No datasets were generated or analysed during the current study.
