# Supplementary figures and images for: Automatic segmentation of male pelvic floor soft tissue structures for anatomical simulation and morphological assessment in lower rectal cancer surgery
Source: Tech Coloproctol. 2025 Oct 8;29(1):176. doi: 10.1007/s10151-025-03218-z (PMC12507984; doi:10.1007/s10151-025-03218-z)

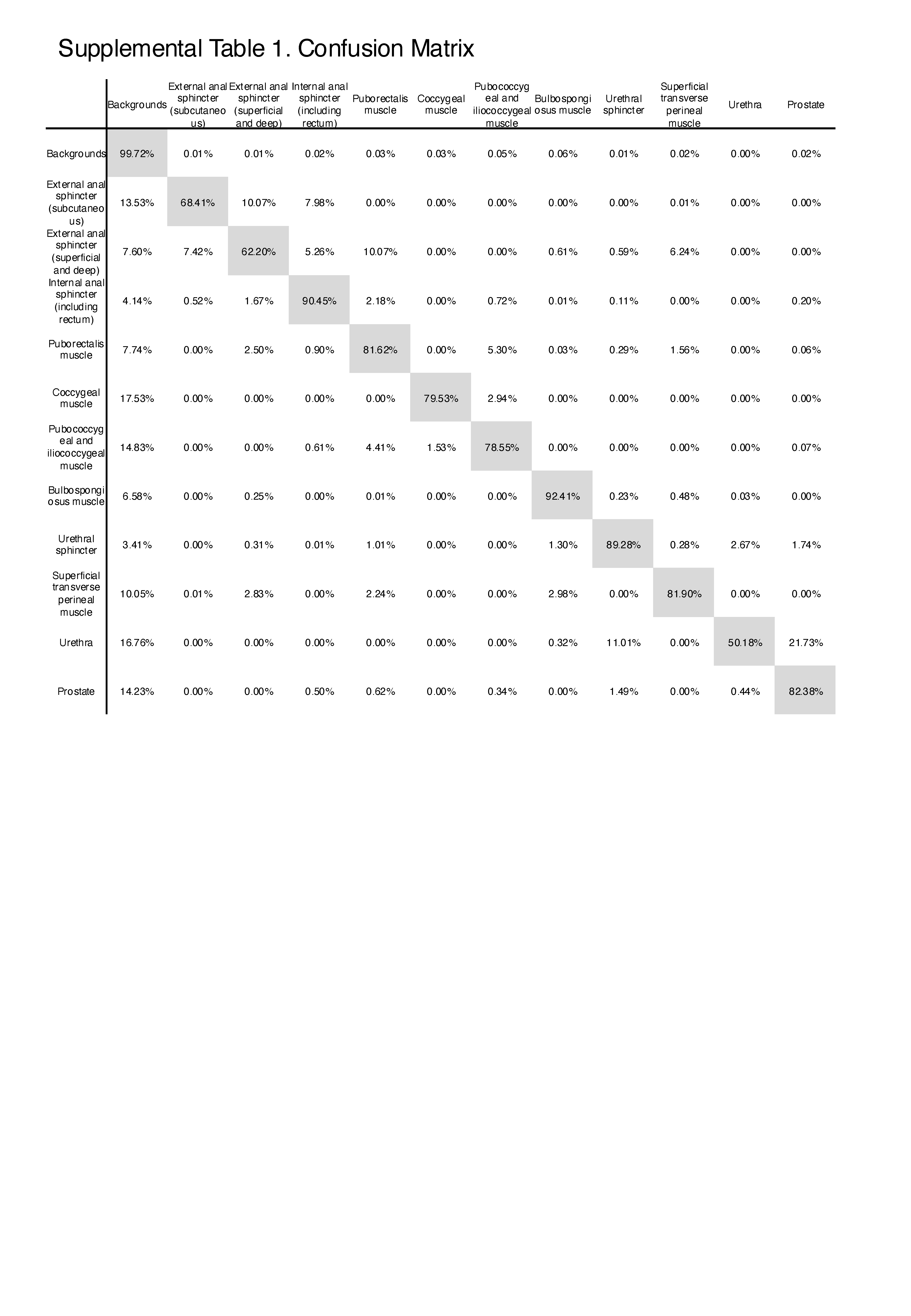

Supplement: Supplementary file 1 — Supplementary file1 (TIFF 946 KB) [file 10151_2025_3218_MOESM1_ESM.tiff]
